# Tumor-to-tumor metastasis of colorectal adenocarcinoma within a gonadotroph pituitary adenoma

**DOI:** 10.1210/jcemcr/luag325

**Published:** 2026-10-22

**Authors:** Shingo Murasawa, Fumiya Isozaki, Miku Fukasawa-Maeda, Shinobu Takayasu, Naoko Inoshita, Yukihiro Fujita

**Affiliations:** Department of Endocrinology and Metabolism, Hirosaki University Graduate School of Medicine, Hirosaki 036-8562, Japan; Department of Endocrinology and Metabolism, Hirosaki University Graduate School of Medicine, Hirosaki 036-8562, Japan; Department of Pathology, Moriyama Memorial Hospital, Tokyo 134-0081, Japan; Department of Endocrinology and Metabolism, Hirosaki University Graduate School of Medicine, Hirosaki 036-8562, Japan; Department of Pathology, Moriyama Memorial Hospital, Tokyo 134-0081, Japan; Department of Endocrinology and Metabolism, Hirosaki University Graduate School of Medicine, Hirosaki 036-8562, Japan

**Keywords:** tumor-to-tumor metastasis, pituitary adenoma, pituitary metastasis, colorectal cancer, hypopituitarism

## Abstract

Pituitary metastases are uncommon, and most cases originate from breast or lung cancer. Tumor-to-tumor metastasis, in which one neoplasm metastasizes into another, is particularly rare in the sellar region. We report a 66-year-old man with metastatic sigmoid colon adenocarcinoma treated with modified FOLFOX6 (5-fluorouracil, L-leucovorin, and oxaliplatin) plus panitumumab; extracranial disease was controlled, whereas a sellar mass initially suspected as a pituitary adenoma slowly enlarged over 2 years. Endocrine testing showed anterior hypopituitarism without arginine vasopressin deficiency. Endoscopic transsphenoidal surgery was performed for continued enlargement and diagnostic uncertainty. Histopathology demonstrated a gonadotroph pituitary adenoma infiltrated by colorectal adenocarcinoma, with extensive intermingling of the 2 components rather than discrete nests, favoring tumor-to-tumor metastasis over a collision tumor. The metastatic component consisted of viable tumor cells without necrosis or convincing treatment effect despite prolonged chemotherapy. This case underscores that metastatic tumor within a pituitary adenoma may enlarge despite favorable systemic therapy outcomes. Metastasis should therefore remain a key differential diagnosis for a sellar mass radiologically suggestive of a pituitary adenoma.

## Introduction

Pituitary metastasis is uncommon and usually occurs in disseminated malignancy, most often from breast or lung cancer [1, 2]. Clinical manifestations include headache, visual symptoms, hypopituitarism, and arginine vasopressin deficiency (AVP-D), since metastatic deposits commonly involve the posterior pituitary and/or pituitary stalk [1, 2]. Tumor-to-tumor metastasis (TTM) is metastatic seeding of one neoplasm into another histologically distinct neoplasm and should be distinguished from collision tumors and direct contiguous invasion [3-5]. Pituitary adenomas can serve as recipient tumors, and colorectal carcinoma metastasis to a pituitary adenoma has been reported [6-8]. Such lesions may mimic a solitary pituitary adenoma, complicating preoperative diagnosis [6-9]. We report TTM of sigmoid colon adenocarcinoma within a gonadotroph pituitary adenoma that enlarged despite extracranial response to systemic therapy.

## Case presentation

A 66-year-old man was initially admitted to another hospital for episodic dizziness and hyponatremia, with a serum sodium concentration of 120 mmol/L (reference range, 138-145 mmol/L). Available records showed an adrenocorticotropin (ACTH) concentration of 5.6 pg/mL (International System of Units [SI]: 1.2 pmol/L; reference range, 7.2-63.3 pg/mL [SI: 1.6-13.9 pmol/L]) and a serum cortisol concentration of 10.8 μg/dL (SI: 298 nmol/L; reference range, 7.07-19.60 μg/dL [SI: 195-541 nmol/L]); thyroid function test results were not available. The hyponatremia improved with intravenous isotonic fluid therapy and oral sodium chloride. No glucocorticoid replacement was prescribed after discharge, and severe hyponatremia did not recur before referral. Subsequent systemic work-up with contrast-enhanced computed tomography (CT) revealed sigmoid colon adenocarcinoma with mediastinal, para-aortic, and pelvic lymph node metastases (stage IVA; Fig. 1A–1D). Bilateral adrenal nodules were also noted on staging CT (Fig. 1E and 1F). While admitted for planned sigmoid colectomy, he experienced recurrent dizziness with near-syncope, prompting head CT that revealed an incidental sellar mass. Because possible hypothalamic–pituitary–adrenal axis impairment could not be excluded preoperatively, colectomy was performed as scheduled with perioperative glucocorticoid replacement. His postoperative course was uncomplicated, and he was referred to our hospital for further evaluation of the sellar lesion and pituitary function. On admission to our department, his blood pressure was 148/91 mmHg. At presentation, he reported no visual complaints, and formal ophthalmologic assessment showed no visual field defects. He had neither polyuria nor polydipsia. However, he was found to have anterior hypopituitarism. After endocrine evaluation, hydrocortisone replacement was continued, and levothyroxine replacement was initiated. Given the absence of overt mass effect and the priority of treating metastatic colorectal cancer, systemic anticancer therapy with modified FOLFOX6 (mFOLFOX6; 5-fluorouracil, L-leucovorin, and oxaliplatin) plus panitumumab was initiated. Oxaliplatin was later discontinued because of peripheral neuropathy, but the systemic therapy was continued. During systemic therapy, the extracranial lesions responded or remained stable. In contrast, the sellar mass gradually enlarged over approximately 2 years, predominantly in the craniocaudal dimension, from 19 to 24 mm on coronal contrast-enhanced T1-weighted magnetic resonance imaging (MRI); the maximal transverse diameter remained unchanged at 31 mm (Fig. 2). Because the lesion enlarged despite extracranial disease control, endoscopic transsphenoidal surgery was pursued for diagnosis and local control.

**Figure 1:**
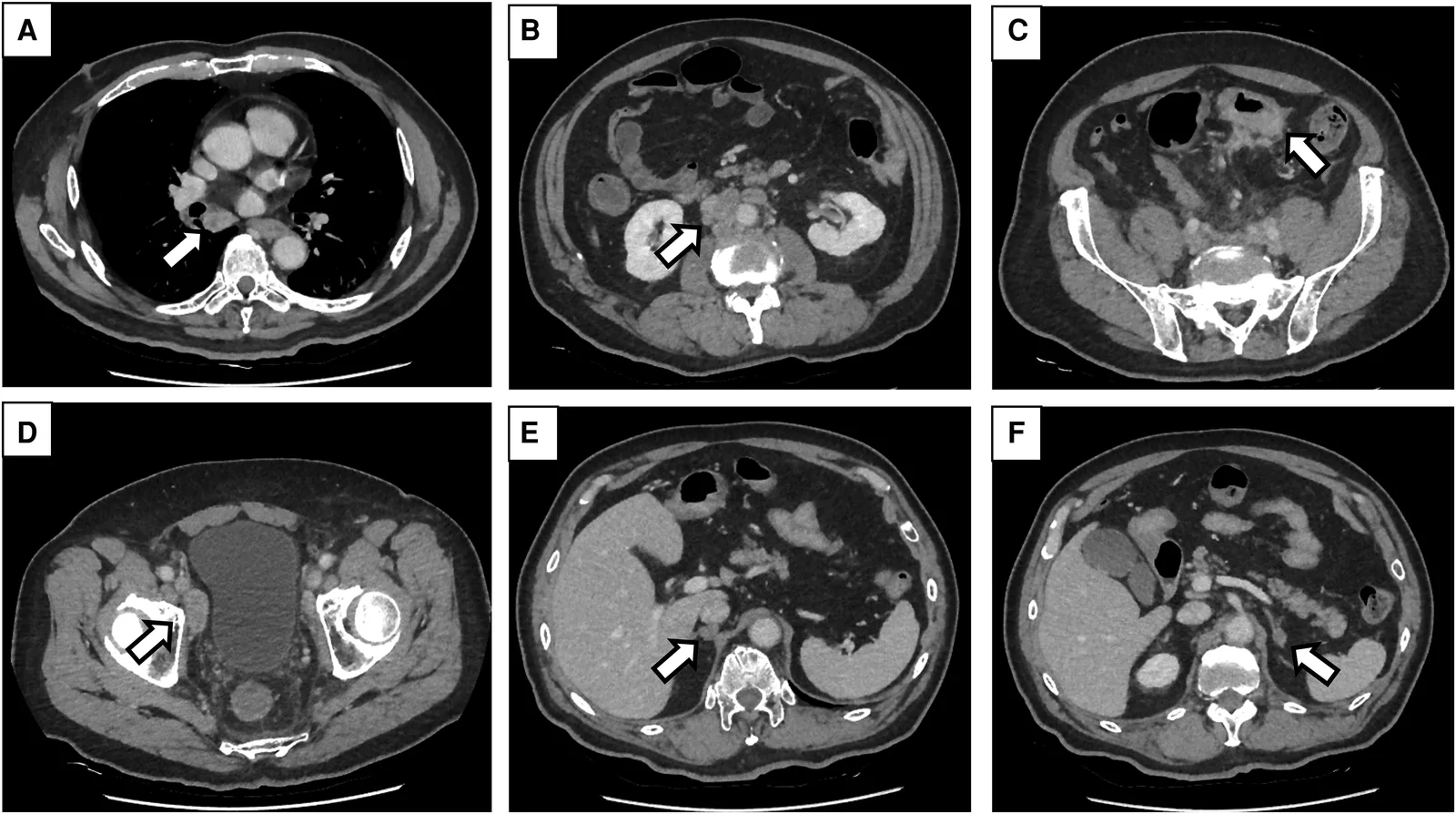
Contrast-enhanced computed tomography at diagnosis. Axial images demonstrate mediastinal lymphadenopathy (A), para-aortic lymphadenopathy (B), the primary sigmoid colon lesion (C), a metastatic external iliac lymph node (D), and bilateral adrenal nodules (right, E; left, F). Arrows indicate the lesions.

**Figure 2:**
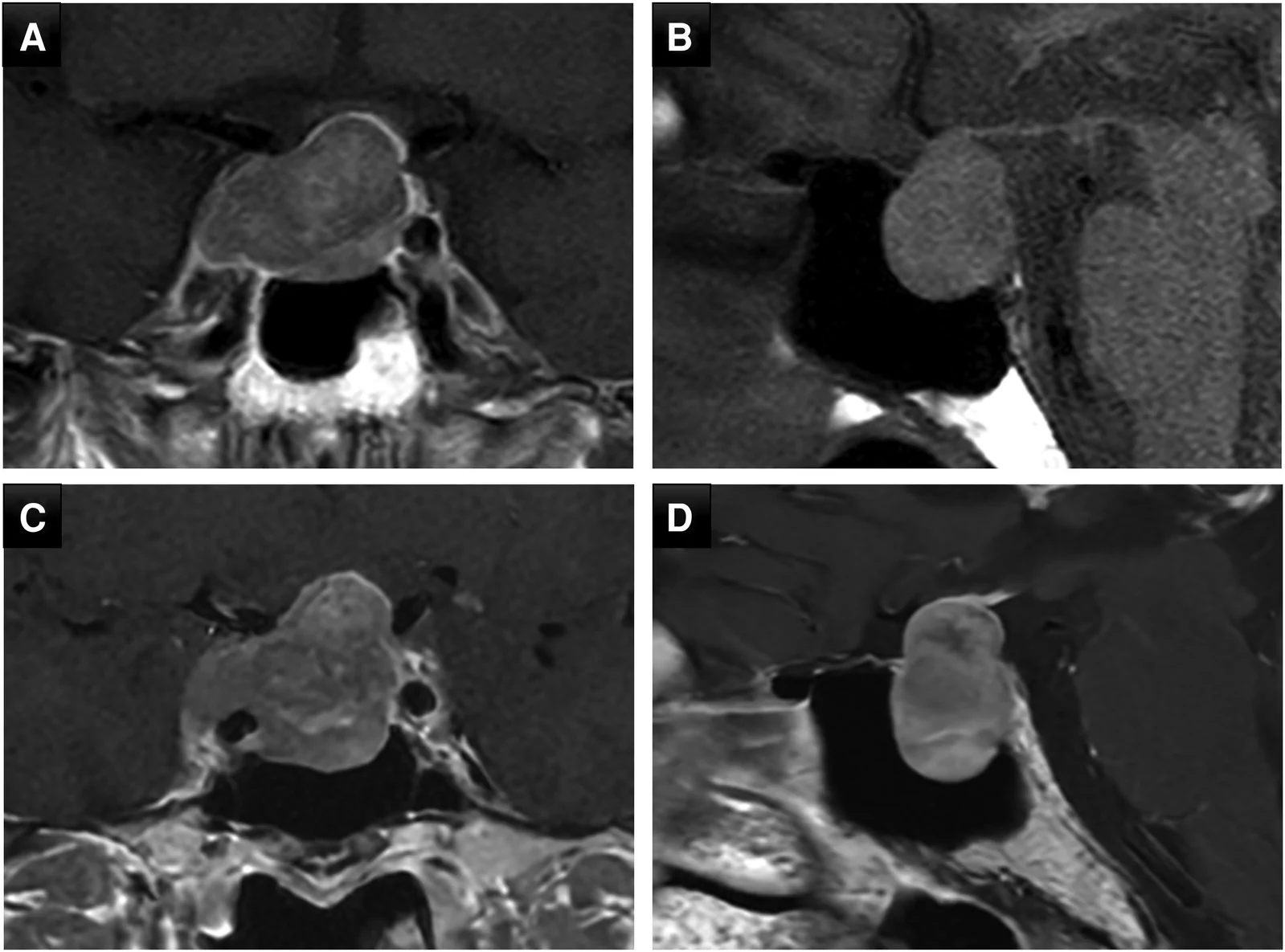
Pituitary magnetic resonance imaging at presentation and before surgery. Contrast-enhanced coronal T1-weighted imaging at initial evaluation (A) shows a sellar mass radiologically consistent with a pituitary adenoma. Sagittal noncontrast T1-weighted imaging at initial evaluation is shown for anatomic reference (B). Approximately 2 years later, in the preoperative period, contrast-enhanced coronal (C) and sagittal (D) T1-weighted images demonstrate slow interval growth. Tumor height increased from 19 to 24 mm, whereas the maximal transverse diameter remained unchanged at 31 mm, measured on coronal contrast-enhanced T1-weighted images.

## Diagnostic assessment

Sellar MRI showed a heterogeneously enhancing mass radiologically consistent with a pituitary adenoma, without features specifically suggestive of metastatic disease (Fig. 2).

Comprehensive endocrine testing before transsphenoidal surgery, including reference ranges, is summarized in Table 1. Thyroid function tests showed markedly low free thyroxine and free triiodothyronine with only a minimally elevated thyroid-stimulating hormone concentration, supporting central hypothyroidism rather than primary hypothyroidism. Dynamic testing also supported anterior hypopituitarism involving the gonadal and growth hormone axes. AVP-D was considered unlikely: the patient reported neither polyuria nor polydipsia, daily urine volume remained <3 L/day, and serum sodium was 141 mmol/L (reference range, 138-145 mmol/L) with plasma vasopressin 0.5 pg/mL (SI: 0.46 pmol/L; reference range, <2.8 pg/mL [SI: <2.6 pmol/L]).

**Table 1:** Endocrine evaluation and dynamic pituitary function testing before transsphenoidal surgery

Parameters	Values	Reference range
Baseline tests		
Serum sodium	141 mmol/L	138 to 145 mmol/L
ACTH	<1.5 pg/mL (<0.33 pmol/L)	7.2 to 63.3 pg/mL (1.6-13.9 pmol/L)
Cortisol	6.5 µg/dL (179 nmol/L)	7.07 to 19.60 µg/dL (195-541 nmol/L)
TSH	5.18 µIU/mL (5.18 mIU/L)	0.50 to 5.00 µIU/mL (0.50-5.00 mIU/L)
Free T4	0.50 ng/dL (6.4 pmol/L)	0.90 to 1.70 ng/dL (11.6-21.9 pmol/L)
Free T3	1.40 pg/mL (2.2 pmol/L)	2.30 to 4.00 pg/mL (3.5-6.1 pmol/L)
Prolactin	13.8 ng/mL (13.8 µg/L)	0.0 to 32.7 ng/mL (0.0-32.7 µg/L)
Total testosterone	6 ng/dL (0.21 nmol/L)	131 to 871 ng/dL (4.55-30.2 nmol/L)
IGF-1	51 ng/mL (6.7 nmol/L)	70 to 219 ng/mL (9.1-28.6 nmol/L)*^a^*
DHEA-S	25 µg/dL (0.68 µmol/L)	24 to 244 µg/dL (0.65-6.62 µmol/L)
Plasma vasopressin	0.5 pg/mL (0.46 pmol/L)	<2.8 pg/mL (<2.6 pmol/L)
Urinary free cortisol (24 hours)	43.2, 14.4, 13.8 µg/day (119, 39.7, 38.1 nmol/day)	11 to 80 µg/day (30-221 nmol/day)
Dynamic endocrine testing		
CRH + GnRH test: ACTH	Basal <1.5 pg/mL (<0.33 pmol/L); peak 18.7 pg/mL (4.12 pmol/L) at 30 and 60 minutes	
CRH + GnRH test: cortisol	Basal 6.5 µg/dL (179 nmol/L); peak 12.7 µg/dL (350 nmol/L) at 90 and 120 minutes	
CRH + GnRH test: LH	Basal 1.2 mIU/mL (1.2 IU/L); peak 3.6 mIU/mL (3.6 IU/L) at 90 and 120 minutes	
CRH + GnRH test: FSH	Basal 3.1 mIU/mL (3.1 IU/L); peak 4.3 mIU/mL (4.3 IU/L) at 120 minutes	
Insulin tolerance test: glucose	Basal 86 mg/dL (4.8 mmol/L); nadir 42 mg/dL (2.3 mmol/L) at 30 minutes	
Insulin tolerance test: ACTH	ACTH remained <1.5 pg/mL (<0.33 pmol/L) throughout	
Insulin tolerance test: cortisol	Basal 5.2 µg/dL (143 nmol/L); peak 6.7 µg/dL (185 nmol/L) at 60 minutes	
Insulin tolerance test: GH	Basal 0.25 ng/mL (0.25 µg/L); peak 0.25 ng/mL (0.25 µg/L) at 0 and 30 minutes	

Basal values correspond to time 0 minutes. Peak values are the highest measured values during each test; peak time indicates the sampling point(s) at which the peak was observed. Values reported as “<1.5” indicate the assay lower limit of quantification. Three 24-hour urinary free cortisol collections are shown.

Abbreviations: ACTH, adrenocorticotropin; CRH, corticotropin-releasing hormone; DHEA-S, dehydroepiandrosterone sulfate; FSH, follicle-stimulating hormone; GH, growth hormone; GnRH, gonadotropin-releasing hormone; IGF-1, insulin-like growth factor 1; LH, luteinizing hormone; T3, triiodothyronine; T4, thyroxine; TSH, thyroid-stimulating hormone.

^*a*^IGF-1 reference range is age- and sex-specific (as provided in the institutional report).

Screening for adrenal hormone excess did not strongly suggest primary aldosteronism or pheochromocytoma. Plasma renin activity was 0.8 ng/mL/hour (SI: 0.8 μg/L/hour; reference range, 0.2-2.3 ng/mL/hour [SI: 0.2-2.3 μg/L/hour]), plasma aldosterone concentration was 82.4 pg/mL (SI: 229 pmol/L; reference range, 4.0-82.1 pg/mL [SI: 11.1-228 pmol/L]), and the aldosterone–renin ratio was 103, below the institutional screening cutoff of 200. Urinary metanephrine was 0.05 mg/g creatinine (SI: 0.029 μmol/mmol creatinine; reference range, 0.04-0.18 mg/g creatinine [SI: 0.023-0.103 μmol/mmol creatinine]), and urinary normetanephrine was mildly elevated at 0.32 mg/g creatinine (SI: 0.198 μmol/mmol creatinine; reference range, 0.10-0.28 mg/g creatinine [SI: 0.062-0.173 μmol/mmol creatinine]). Interpretation of the hypothalamic–pituitary–adrenal axis required caution because of glucocorticoid replacement, pituitary dysfunction, and bilateral adrenal nodules. After colectomy, hydrocortisone was continued at 15 mg/day and reduced to 10 mg once daily after breakfast at admission to our department; endocrine testing in Table 1 was performed during this replacement therapy. For 24-hour urinary free cortisol collections, hydrocortisone was replaced with dexamethasone 0.25 mg/day to avoid assay cross-reactivity; urinary free cortisol values were low-normal but within the reference range. Adrenocorticotropin was below the assay sensitivity limit, and the blunted response to corticotropin-releasing hormone suggested impaired corticotroph reserve due to the sellar lesion, although suppression from autonomous cortisol secretion may also have contributed. A 1-mg dexamethasone suppression test before the morning hydrocortisone dose showed inadequate cortisol suppression (9.0 μg/dL [SI: 248 nmol/L]; diagnostic threshold, ≥5.0 μg/dL [SI: ≥138 nmol/L]), fulfilling the Japanese diagnostic criteria for adrenal subclinical Cushing syndrome, broadly corresponding to mild autonomous cortisol secretion [10]. Because the adrenal nodules were not pathologically confirmed, metastatic disease could not be ruled out.

Histopathology of the resected sigmoid colon tumor showed a moderately differentiated adenocarcinoma with a poorly differentiated component on hematoxylin–eosin staining (Fig. 3). Histopathology of the resected sellar mass demonstrated 2 intimately admixed components (Fig. 4). One component comprised a relatively uniform population of small round cells with pale eosinophilic cytoplasm arranged in solid sheets, compatible with pituitary adenoma. The other component consisted of atypical columnar cells forming irregular glands without necrosis or convincing treatment effect. On low power, CDX2 immunostaining highlighted an insular-to-scattered distribution of adenocarcinoma comprising approximately 30% of the tumor area (Fig. 4A); at higher magnification, adenocarcinoma glands were interspersed within the pituitary adenoma background rather than forming discrete metastatic nests with a sharp interface (Fig. 4B and 4C), favoring TTM over a collision tumor.

**Figure 3:**
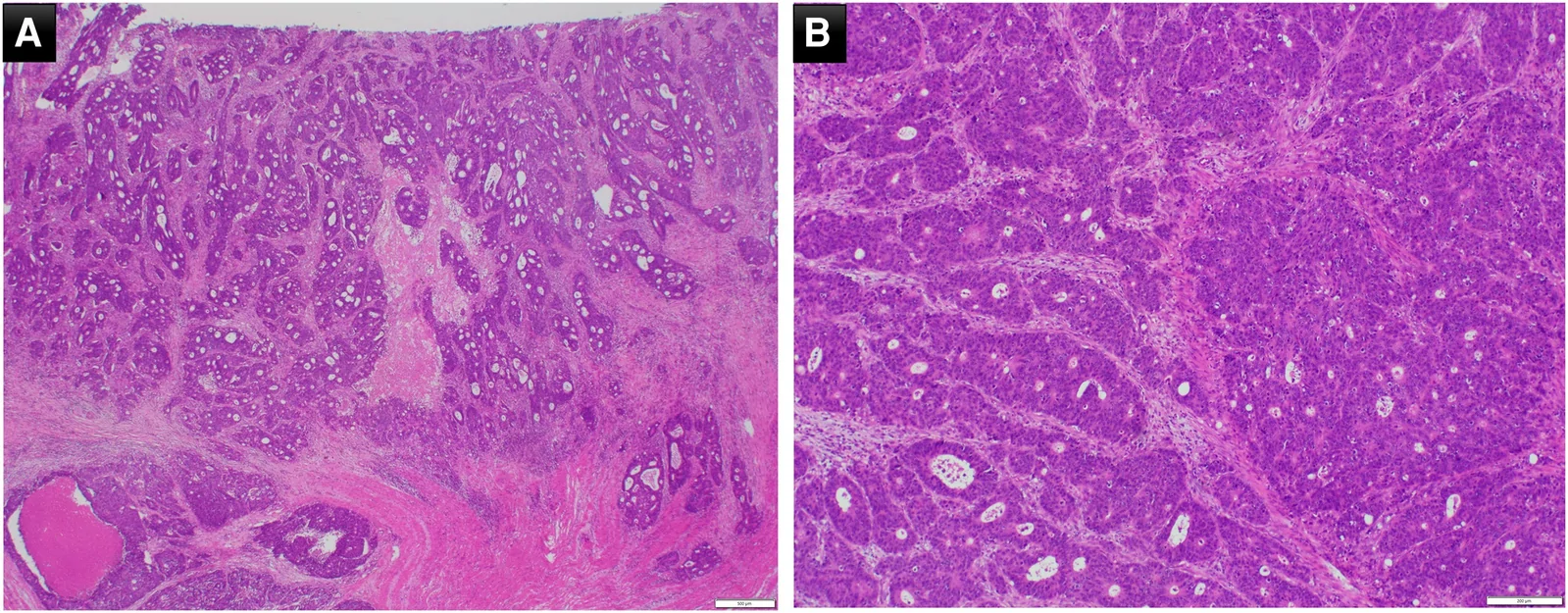
Histopathology of the primary sigmoid colon lesion. (A) Low-power hematoxylin–eosin (H&E) stain shows an invasive, moderately differentiated adenocarcinoma with irregular fused glands infiltrating through the bowel wall. (B) High-power H&E stain demonstrates atypical glandular structures lined by malignant columnar cells. Scale bars, 500 μm (A) and 200 μm (B).

**Figure 4:**
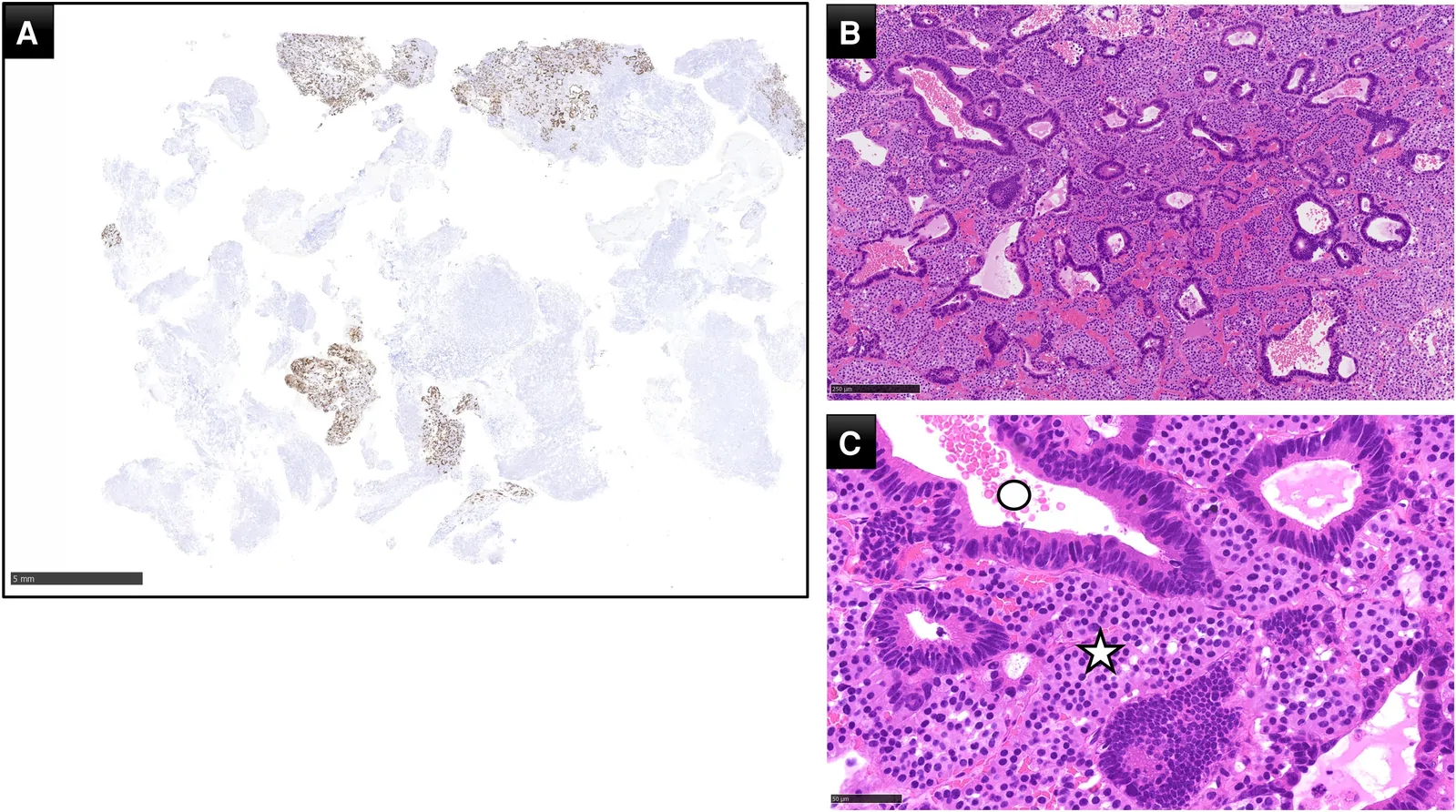
Histopathology of the sellar mass showing colorectal adenocarcinoma within a gonadotroph pituitary adenoma. (A) CDX2 immunohistochemistry highlights metastatic colorectal adenocarcinoma, comprising approximately 30% of the tumor area. (B) Hematoxylin–eosin (H&E) stain shows intimate intermingling of adenocarcinoma glands with the pituitary adenoma component. (C) High-power H&E demonstrates adenocarcinoma glands admixed with monotonous pituitary adenoma cells, without overt necrosis or convincing treatment effect. Scale bars, 5 mm (A), 250 μm (B), and 50 μm (C). In (C), the circle indicates an adenocarcinoma gland, and the star indicates the pituitary adenoma component.

Immunohistochemistry further confirmed the lineage of each component. The adenocarcinoma component was CDX2-positive and SATB2-positive with a CK20-positive/CK7-negative profile (Fig. 5), consistent with colorectal origin, whereas the pituitary adenoma component was steroidogenic factor 1–positive and follicle-stimulating hormone β-subunit–positive, consistent with a gonadotroph pituitary adenoma (Fig. 6). The Ki-67 labeling index was 0.7% in the pituitary adenoma component.

**Figure 5:**
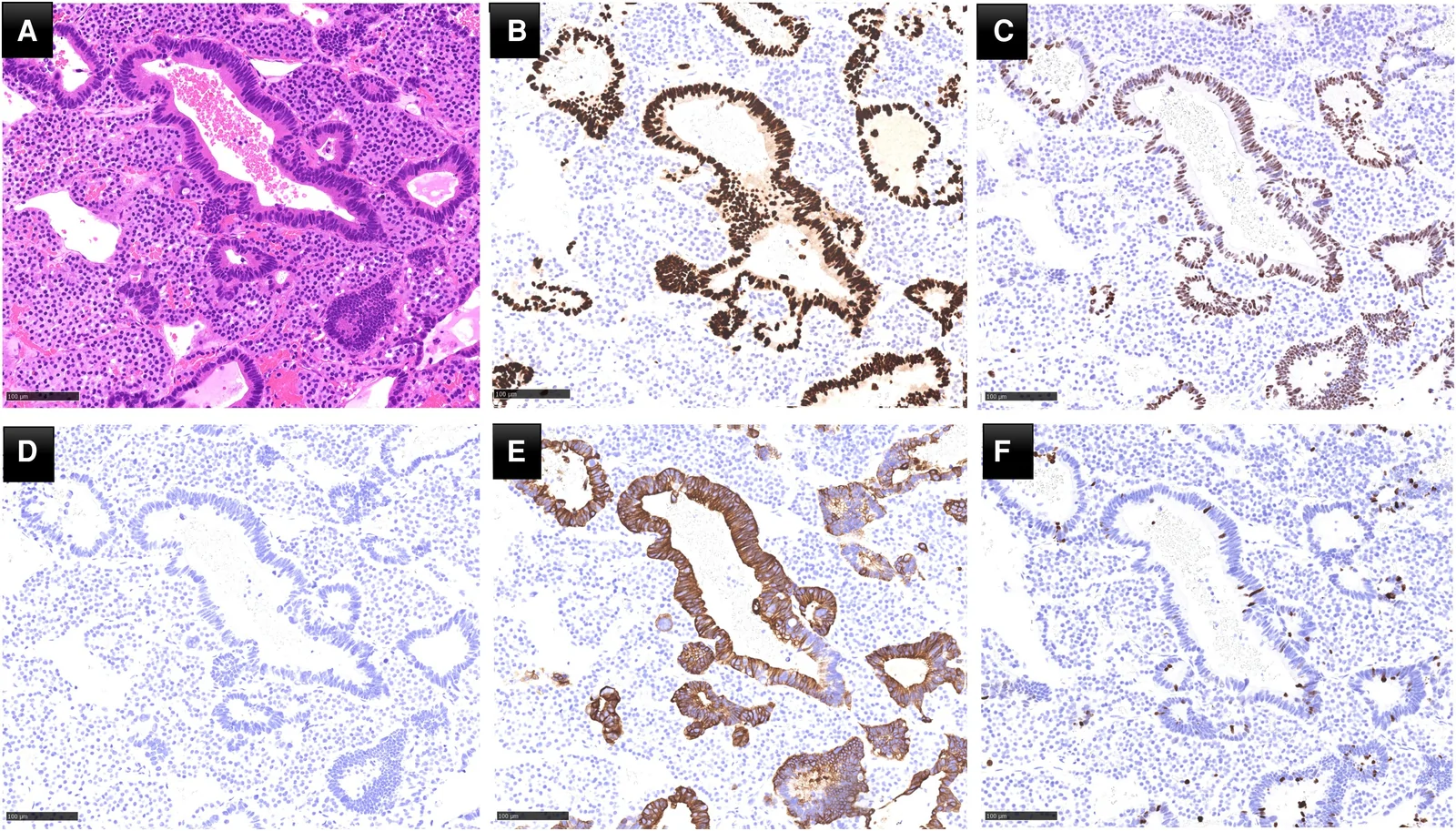
Immunohistochemical profile of the adenocarcinoma component within the sellar lesion. (A) Hematoxylin–eosin (H&E) stain. The adenocarcinoma glands show nuclear positivity for CDX2 (B) and SATB2 (C) and cytoplasmic positivity for CK20 (E), whereas CK7 is negative (D), supporting colorectal origin. (F) Ki-67 immunostaining shows proliferative activity in the adenocarcinoma component. Scale bars, 100 μm (A–F).

**Figure 6:**
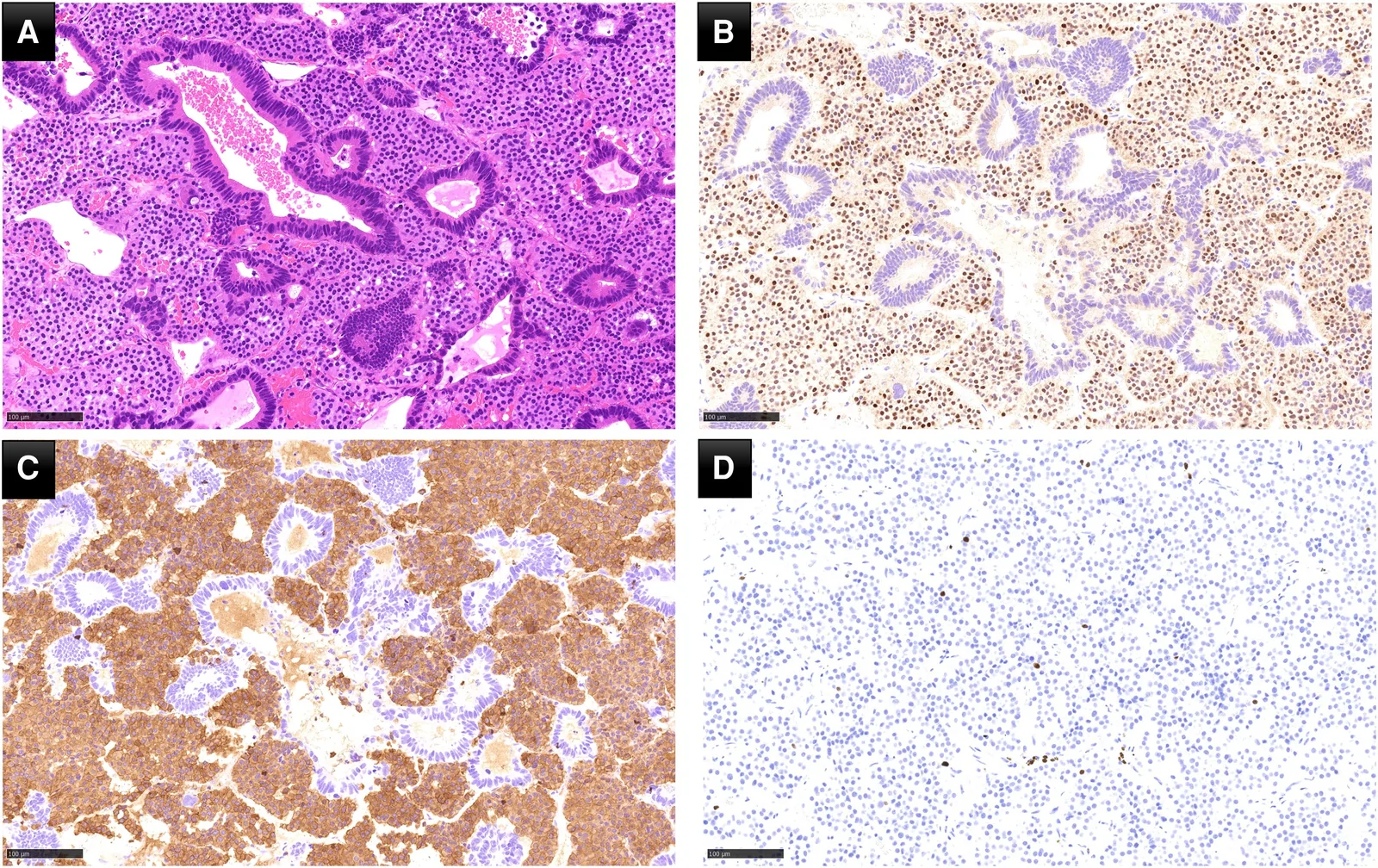
Immunohistochemical confirmation of the gonadotroph pituitary adenoma component. (A) Hematoxylin–eosin (H&E) stain of the pituitary adenoma component shows a relatively uniform population of small round cells with a trabecular/solid growth pattern and focal perivascular arrangement. Immunohistochemistry demonstrates nuclear steroidogenic factor 1 (SF-1) positivity (B) and cytoplasmic follicle-stimulating hormone β-subunit (β-FSH) positivity (C), consistent with gonadotroph differentiation. (D) Ki-67 immunostaining shows a labeling index of 0.7% in the pituitary adenoma component. Scale bars, 100 μm (A–D).

## Treatment

Endoscopic transsphenoidal surgery was performed for debulking and histopathologic diagnosis. Postoperatively, the patient did not develop AVP-D, and daily urine output remained stable. Anterior pituitary function did not recover, and he continued hormone replacement therapy with hydrocortisone and levothyroxine. Testosterone replacement was deferred because of advanced metastatic colorectal cancer, ongoing systemic therapy, and the absence of severe symptoms requiring immediate treatment.

Because residual sellar tumor was present, stereotactic radiosurgery with Gamma Knife was initiated 2 months after transsphenoidal surgery while systemic therapy for colorectal cancer was continued.

## Outcome and follow-up

After Gamma Knife radiosurgery, follow-up imaging showed no regrowth of the residual sellar lesion. The patient continues systemic therapy for metastatic colorectal cancer and remains on hydrocortisone 10 mg/day and levothyroxine 100 µg/day. Anterior pituitary function, including the gonadal axis, has not recovered, testosterone replacement has not been initiated, and no delayed-onset AVP-D has been observed.

## Discussion

This case illustrates TTM of colorectal adenocarcinoma within a gonadotroph pituitary adenoma. Although this tumor combination has been reported [8], the main contribution of our case is the discordant behavior during systemic therapy: extracranial disease was controlled, whereas the sellar lesion enlarged gradually over approximately 2 years. Donofrio et al [8] reported a similar discordance, but with rapid sellar growth during capecitabine therapy.

In the present case, slow enlargement without imaging features suggestive of metastasis precluded radiological distinction between an ordinary pituitary adenoma and one harboring metastatic carcinoma.

A key pathological issue is distinguishing TTM from a collision tumor. By definition, collision tumors originate as adjacent but separate neoplasms, whereas TTM requires metastatic seeding within a histologically distinct recipient tumor [3-5]. In our specimen, intimate admixture of colorectal adenocarcinoma within the pituitary adenoma favored TTM. However, with progression, a collision tumor may also show secondary intermingling, and definitive distinction may not always be possible from a single surgical specimen. Although the immediate therapeutic implications are uncertain, the distinction may be relevant to treatment response within the recipient tumor microenvironment and local treatment planning when the components are spatially separable.

The sellar lesion enlarged gradually over approximately 2 years. Because histopathologic evaluation was limited to 1 surgical specimen, the relative contribution of the pituitary adenoma and metastatic colorectal components remains uncertain. The discordant response during mFOLFOX6 plus panitumumab is notable because the regimen targeted extracranial colorectal cancer, and efficacy against sellar metastasis cannot be inferred from the extracranial response. Heterogeneous blood–tumor barrier permeability may contribute to discordant drug delivery in brain metastases [11], but this explanation is incomplete in the sellar region, which differs from brain parenchyma and includes structures with relatively high vascular permeability [12]. Other possible contributors include clonal heterogeneity across metastatic deposits [13] and the recipient tumor microenvironment, including adenoma-specific vascular architecture, stromal and cytokine milieu, and interactions with pituitary adenoma cells [14]. Viable metastatic carcinoma without necrosis or convincing treatment effect supports true discordance between the sellar lesion and extracranial disease.

Given discordant sellar growth despite extracranial disease control, transsphenoidal surgery was performed for diagnostic confirmation and local control rather than treatment of an ordinary pituitary adenoma. Surgery can provide histopathologic confirmation and symptom relief in pituitary metastasis [15], and previous TTM reports support surgical intervention when diagnosis remains uncertain or local progression is concerning [6-8]. In our case, subtotal resection followed by stereotactic radiosurgery achieved local control while systemic therapy continued.

Finally, the adrenal findings require cautious interpretation. Urinary free cortisol values were low-normal but within the reference range, whereas the 1-mg dexamethasone suppression test, performed before the morning hydrocortisone dose, showed inadequate cortisol suppression and fulfilled the Japanese diagnostic criteria for adrenal subclinical Cushing syndrome, a condition broadly corresponding to mild autonomous cortisol secretion [10]. However, ACTH and cortisol were evaluated in the setting of pituitary dysfunction and glucocorticoid replacement, and the bilateral adrenal nodules were not pathologically confirmed. Thus, possible autonomous cortisol secretion may have contributed to measurable cortisol despite suppressed ACTH, but this endocrine complexity was not central to the diagnosis of TTM.

In summary, pituitary adenoma-like sellar masses in metastatic colorectal cancer may harbor metastatic disease and show selective enlargement despite extracranial disease control.

## Learning points

- Sellar lesions in patients with metastatic colorectal cancer may be radiologically consistent with a pituitary adenoma and enlarge slowly.
- Tumor-to-tumor metastasis to a pituitary adenoma should be considered when a presumed pituitary adenoma shows progressive growth, even without AVP-D.
- Histopathology may show an insular-to-scattered metastatic pattern with intimate admixture, favoring TTM over collision.
- A pituitary adenoma harboring colorectal adenocarcinoma metastasis may show selective sellar enlargement despite extracranial disease control during systemic therapy.

## Data Availability

Original data generated and analyzed for this case report are included in this published article.
